# Social Capital and Economic Growth: An Analysis in South America

**DOI:** 10.12688/f1000research.166766.2

**Published:** 2025-09-08

**Authors:** Aracelly Fernanda Núñez-Naranjo, Ximena Morales-Urrutia

**Affiliations:** 1 Centro de Investigación en Ciencias Humanas y del Educación-CICHE. Facultad de Ciencias de la Educación, Universidad Tecnologica Indoamerica, Ambato 180103, Tungurahua, Ecuador; 2 Facultad de Contabilidad y Auditoría, Universidad Tecnica de Ambato, Ambato, Tungurahua, Ecuador

**Keywords:** economic development, civic engagement, regional disparities, public policy, sustainable growth.

## Abstract

**Background:**

Social capital has emerged as a key variable in explaining regional economic disparities, yet its multidimensional nature complicates measurement and policy design. In the South American context, characterized by institutional fragility and inequality, the impact of social capital on economic growth remains empirically ambiguous.

**Methods:**

This study employs a quantitative methodology using panel data from 2007 to 2023 across nine South American countries. Data is sourced from the Legatum Prosperity Index, focusing on five elements of social capital: family relationships, social networks, interpersonal trust, social tolerance, and civic participation. Econometric analysis is conducted using instrumental variables and two-stage least squares models to address endogeneity.

**Results:**

The econometric findings reveal heterogeneous effects among dimensions of social capital. Civic participation and family relationships exhibit a significant positive association with economic growth, while social tolerance and interpersonal trust show negative or inconsistent impacts. Notably, social networks instrumentalized in the model have a negative and significant relationship with growth, suggesting potential inefficiencies in their current form. The presence of endogeneity and multicollinearity among variables highlights the complexity of causality in this domain.

**Conclusions:**

Social capital plays a critical, yet context-dependent, role in fostering economic development in South America. While some dimensions such as civic engagement promote inclusive growth, others may reinforce exclusion or inefficiencies depending on institutional quality and social structure. Policymakers should therefore target specific aspects of social capital, promoting civic participation and inclusive networks while addressing institutional weaknesses. Future research should explore longitudinal dynamics and the interaction of social capital with digitalization and labor market transformation.

## Introduction

Empirical and theoretical literature has shown that social capital, understood as the network of relationships, trust and cooperation between individuals and organizations, plays a fundamental role in driving economic development. Recent research has shown that these dimensions facilitate access to resources, stimulate innovation and strengthen institutional resilience, all essential elements for the sustainable growth of economies. ^1^ In the South American context, several studies have highlighted how these social interactions translate into higher levels of competitiveness and efficiency in the implementation of economic policies. ^2^

However, despite these advances, there are still significant gaps in the understanding of the underlying mechanisms that mediate the relationship between social capital and economic development in the region. Most theoretical and empirical models have been developed in contexts other than South America, omitting the complexity of cultural, political and structural variables specific to this area. ^3^ Moreover, it is still uncertain how the processes of social capital formation interact and feed back into economic growth strategies, which justifies the need for research that integrates these dimensions in a holistic manner. ^4– 6^

The relationship between social capital and economic growth has been the subject of debate in the academic literature, particularly with regard to the direction of causality. On the one hand, some studies argue that social capital is a determinant of economic growth, as it facilitates cooperation, reduces transaction costs and promotes the trust necessary for the development of efficient markets. ^7, 8^ From this perspective, strong social networks and high levels of interpersonal trust can boost investment, innovation and economic efficiency, especially in contexts where formal institutions are weak. ^2^

On the other hand, there is evidence to suggest that economic growth can be a precursor of social capital. In more prosperous economies, individuals have greater resources and opportunities to participate in social networks and civic activities, which strengthens the social fabric. ^9^ In addition, economic development can generate more inclusive and transparent institutions, which in turn foster trust and cooperation among citizens. ^10^ This bidirectionality in the relationship between social capital and economic growth poses a methodological challenge for empirical studies, as it is difficult to establish clear causality without considering feedback effects between both variables.

In the South American context, this discussion takes on particular relevance due to the structural inequalities and institutional weaknesses that characterize the region. For example, in countries such as Brazil and Colombia, high levels of economic inequality have limited the capacity of social networks to generate inclusive economic benefits, while in Uruguay, greater equity has allowed for a strengthening of social capital and more sustainable economic growth. ^11^ Therefore, it is crucial to address this theoretical controversy in the analysis, recognizing that the relationship between social capital and economic growth may vary depending on the socioeconomic and institutional context.

The measurement of social capital has been a persistent challenge in economic and social research due to its multidimensional and subjective nature. Despite the increasing attention it has received in recent years, the lack of methodological consensus has led to a proliferation of contradictory findings, which complicates its applicability in the design of public policies and economic development strategies.

Although social capital is usually associated with positive effects, it can also generate negative externalities, especially in contexts of high inequality and social exclusion. One of the most recurrent criticisms is that social capital can be exclusionary, i.e., that social networks can benefit certain groups while marginalizing others. For example, in communities with closed and homogeneous networks, strong ties can reinforce existing hierarchies and limit access to economic resources for those who are not part of these networks. ^12^ This phenomenon is particularly relevant in South America, where social networks often reflect ethnic, racial and class divisions, perpetuating the exclusion of historically marginalized groups.

In addition, social capital can limit innovation by favoring conformity and discouraging critical thinking. In contexts where social networks are highly cohesive, the pressure to maintain group harmony can discourage diversity of ideas and competition, key elements for innovation and economic growth. ^12^ For example, in some rural communities in Bolivia and Peru, traditional social networks have hindered the adoption of new agricultural technologies, limiting local economic development. ^2^

Social capital can also be associated with corrupt or clientelistic practices, especially in contexts where institutions are weak. In some South American countries, social networks have been used to facilitate privileged access to public resources, perpetuating corruption and eroding trust in institutions. ^13^ These examples illustrate the need to address the possible negative externalities of social capital in the analysis, recognizing that their effects are not always positive and that they depend to a large extent on the context in which they develop.

One of the main problems in measuring social capital lies in the absence of a single, universally accepted indicator. ^14^ Unlike other economic variables, such as GDP or the employment rate, social capital is expressed through interpersonal relationships, shared norms and trust networks, aspects that are difficult to quantify. The literature has employed various approaches, such as perception surveys, social network analysis, and the use of proxies such as participation in civic organizations or the density of community associations. ^15, 16^ However, these methods present inherent limitations, such as social desirability bias in surveys or the inability of proxies to capture the richness of the concept.

On the other hand, social capital has been conceptualized from different theoretical traditions that have generated debates on its nature, measurement and impact on economic development. From an individualistic perspective, linked to the approaches of, ^17, 18^ social capital is conceived as a resource accumulated by individuals through their interpersonal relationships, where trust and reciprocity facilitate access to economic opportunities. ^14, 19, 20^ This approach emphasizes the role of personal networks and investment in relationships as mechanisms to reduce transaction costs and improve the efficiency of markets. ^7, 21^ However, its emphasis on individual agency has been criticized for not adequately considering the social structures that condition the generation and distribution of social capital. ^5^

On the other hand, the structural approach, derived from social networks and structural sociology, argues that social capital does not reside solely in individuals, but in the configuration and density of ties within a community. Recent studies have shown that social cohesion and network density strengthen the capacity of local economies to withstand economic crises and foster innovation. ^6^

In contrast to these approaches, the institutionalist perspective has gained relevance in the last decade by highlighting the interaction between social capital and the political and normative structures that regulate economic life. ^10, 22^ From this perspective, social capital is not an asset exclusively generated by individuals or networks, but is conditioned by the quality of institutions that promote trust, contract enforcement and the reduction of corruption. Recent research in South America has suggested that institutional weakness and structural inequality can erode the positive effects of social capital on economic growth, evidencing the need to approach its analysis from a more systemic approach. ^13^

The main objective of this research is to analyze the impact of the various dimensions of social capital on economic development in South American countries. To this end, a quantitative methodological approach was adopted, using regression models with panel data. This contribution not only enriches the academic debate but also guides policy makers and social actors in the implementation of initiatives that enhance social capital as a driver of development.

## Background

### Origin and development of Social Capital

The term “Social Capital” was originally used as a metaphor to describe activities that strengthen community cohesion, such as social gatherings for constructive purposes. ^23, 24^ Although this view laid the theoretical foundation, CS remained underdeveloped as an academic concept until the contributions of authors such as Coleman and Bourdieu.

Reference [ 25] defined CS as a resource derived from interpersonal relationships within a social structure, highlighting how these connections enable the achievement of individual and collective goals. According to this approach, CS emerges from the actions of individuals interested in maximizing personal benefits within an established normative framework. ^26^

On the other hand, ^27^ introduced a more structuralist perspective, arguing that SC depends on the tangible and intangible resources that groups and communities use to improve their living conditions. According to Bourdieu, the transfer of intangible resources, such as trust and social cohesion, is crucial to ensure the continuity of economic and social development between generations.

Reference [ 28] expanded the concept to include structural features such as norms, social networks and trust, which facilitate cooperation for collective benefit. In addition, he stressed that CS generates positive externalities, not only for the individuals involved, but for society as a whole, which makes it a strategic resource for economic development.

### Theories underpinning Social Capital

The social capital theory of Bourdieu ^27^ and Coleman ^26^ who conceive this concept as a resource accumulated by individuals through their interpersonal relationships. Bourdieu ^17^ defines social capital as the set of current and potential resources linked to the possession of a durable network of more or less institutionalized relationships of recognition and reciprocity. ^29^ In this framework, social capital is an asset that individuals can use to improve their socioeconomic position, facilitating access to economic, employment and educational opportunities, ^25^ for his part, conceptualizes it as a structure of relationships that favors cooperation and efficiency in collective action, allowing the reduction of transaction costs and the optimization of economic behavior. This approach has been criticized for its emphasis on individual agency, leaving aside structural constraints and the role of institutions in the formation of social capital. ^30, 31^

From a structural perspective, social capital is understood as an emergent phenomenon of interaction within social networks, where the density of ties and group cohesion determine its impact on economic development. ^32– 35^ Introduced the notion of “the strength of weak ties,” arguing that indirect connections may be more valuable than close relationships for accessing information and economic resources. ^36, 37^ Building on this approach, ^38^ develops the theory of “structural holes,” emphasizing that the position of an individual or entity within a network influences its ability to generate value through social capital. This perspective emphasizes that the pre-existing social structure conditions access to economic opportunities and entrepreneurial success, as dense networks favor trust and cooperation, while fragmented networks can limit the flow of information and resources. ^2^ In South America, recent studies have shown that the cohesion of community networks impacts the economic resilience of vulnerable sectors, reinforcing the relevance of structural social capital in contexts of inequality and social exclusion. ^39^

In the South American context, social capital is deeply influenced by historical inequalities and institutional weakness. ^12^ For example, in countries such as Brazil and Colombia, social networks have been used both to foster community cooperation and to perpetuate clientelistic practices that reinforce the exclusion of marginalized groups. ^40^ In addition, lack of trust in public institutions, resulting from high levels of corruption and lack of transparency, has eroded the capacity of social capital to generate inclusive economic benefits. ^41, 42^ In contrast, countries such as Uruguay have managed to strengthen social capital through public policies that promote equity and citizen participation, which has had a positive impact on their economic development. ^43^

For his part, ^44^ argues that formal institutions (laws, regulations) and informal institutions (norms, cultural values) condition the accumulation of social capital and, therefore, its impact on economic development. From this perspective, ^10^ argue that social capital flourishes in societies with inclusive institutions, where norms guarantee equitable participation in economic activity and reduce incentives for opportunism and corruption (ECLAC 2022). In South America, institutional weakness has been identified as a factor limiting the positive impact of social capital on economic growth, as lack of trust in public institutions and high perceptions of corruption can erode cooperative networks. ^45^ This approach highlights the need to consider the interaction between social networks and institutional structures to understand the true potential of social capital in the region’s economic development. ^46^

In this regard, the measurement of social capital represents a significant methodological challenge due to its multidimensional nature and its reliance on difficult-to-measure constructs, such as trust, social networks and norms of reciprocity. The lack of consensus on a universal definition has led to a proliferation of divergent approaches, from survey-based indicators to observable behavioral metrics. ^47^ While some studies have adopted subjective measures, such as perception surveys on interpersonal trust and civic engagement, others have turned to objective approaches, such as associationism rates or network density. However, these differences in measurement generate comparative biases and limit the external validity of the studies, making it difficult to generalize the results. ^48^ Moreover, endogeneity remains a persistent problem, as causal relationships between social capital and economic growth may be mediated by omitted factors, compromising the robustness of empirical findings. ^24, 49^

In this context, reconciling contradictory findings in the literature requires a more systematic approach. There are discrepancies that can be attributed to variations in institutional and cultural contexts, as well as differences in levels of analytical aggregation (individual, community or national). The heterogeneity in the effects of social capital suggests that its impact is neither homogeneous nor linear, but depends on complementary factors, such as the quality of institutions and human capital development. ^50^

### Relationship between Social Capital and economic growth

From a theoretical perspective, social capital is linked to economic growth through three fundamental mechanisms: the reduction of transaction costs, the generation of trust and the promotion of cooperation in economic activity. ^35^ At the macroeconomic level, social trust and reciprocity have been identified as determinants of market stability and investment attraction. ^51, 52^ From an empirical approach, various research has shown that countries with higher levels of social capital tend to exhibit more sustained and equitable economic growth. Recent studies have found a positive correlation between interpersonal trust and Gross Domestic Product (GDP) per capita in emerging economies, suggesting that greater social cohesion boosts productive efficiency and innovation. ^53^

In the specific case of Latin America, social capital can be a key factor in facing economic crises and promoting more inclusive growth models. Recent research suggests that strengthening social capital in local communities favors the implementation of more effective and sustainable public policies by encouraging citizen participation and co-responsibility in resource management. ^54^ Thus, experiences such as those of Brazil and Chile have shown that the strength of community networks can compensate for institutional deficits, fostering cooperation between the private and public sectors to improve economic governance. However, to maximize these positive effects, it is essential to complement social capital with institutional reforms that guarantee transparency and reduce corruption. In this way, social capital would not only contribute to economic growth, but also strengthen democratic stability and social cohesion in the region, consolidating a solid foundation for sustainable and equitable development ^55, 56^

## Methodology

A quantitative approach was chosen because it allows for the establishment of causal relationships and the measurement of the magnitude of the impact of the different dimensions of social capital on economic growth. The use of panel data (2007–2023) is advantageous as it combines cross-sectional and temporal information, which increases statistical robustness and makes it possible to capture long-term dynamics while reducing bias derived from unobserved heterogeneity.

This research uses a quantitative approach and is based on data from the ^57^ which covers information from 167 countries, representing more than 99% of the world’s population. This index offers a comprehensive view of the social, economic and cultural characteristics of nations, providing a solid basis for assessing the relationship between social capital and economic growth in South American countries.

The index is structured in three domains, 12 pillars and 67 elements. The “Inclusive Societies” domain includes the Social Capital pillar, measured by five elements that are family and personal relationships, social networks, interpersonal trust, social tolerance, and social and civic participation.

On the other hand, the “Open Economies” domain included the Economic Quality pillar, which evaluates aspects such as fiscal sustainability, macroeconomic stability, productivity, competitiveness and labor participation.

The analysis focuses on nine South American countries: Argentina, Bolivia, Brazil, Chile, Colombia, Ecuador, Paraguay, Peru and Uruguay. These countries were selected because they present relatively stable conditions, allowing an effective comparison and a robust interpretation of the results. Countries such as Guyana and Suriname were excluded because their small economies and scattered data make a representative analysis difficult. In addition, Venezuela, due to the political, social and economic crisis in the country, generates instability in the data, making a homogeneous comparison with the rest of the region impossible.

The final sample was therefore limited to these nine countries because they provide consistent and comparable data for the 2007–2023 period and represent the largest and most relevant economies in South America, with institutional structures that allow for a robust interpretation of the findings. The exclusion of other cases was not arbitrary but based on the need to ensure data quality, homogeneity, and analytical validity.

The study combines a descriptive and analytical approach, with the following methodological steps.

### Descriptive analysis

The main characteristics of the elements of social capital in each country are identified and described. This analysis makes it possible to interpret trends and changes over the period studied (2007-2023), providing a comparative overview of the elements in the different countries.

### Econometric analysis

To analyze the relationship between social capital and economic growth, an econometric approach based on instrumental variables models will be used. First, the Durbin-Wu-Hausman test will be carried out to detect possible endogeneity problems in the explanatory variables, particularly in the measures of social capital. In the case that the presence of endogeneity is confirmed, estimation will proceed by means of a Two-Stage Ordinary Least Squares Model. In the first stage, the selected instrumental variables will be used to estimate the predicted values of the endogenous variables. In the second stage, these predicted values will be incorporated into the structural equation of economic growth to obtain unbiased and consistent estimates.

## Results

### Description of the behavior of the Social Capital of South American countries in the period 2007-2022.

**Figure 1.**
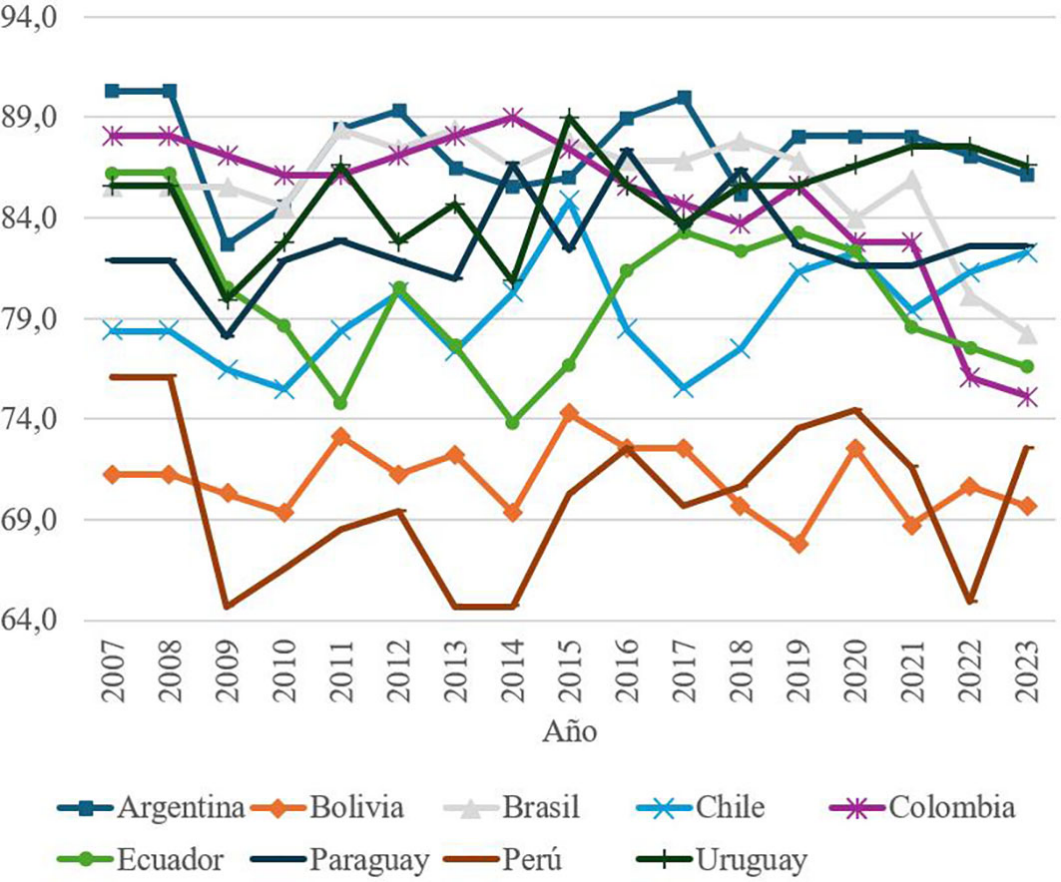
Evolution of social and family relationships by country, 2007–2023.

The first element of social capital, called Social and Family Relations, is composed of two indicators: the percentage of people who feel that they can count on the help of friends and family in case of problems, and the percentage of people who perceive that their family environment transmits positive energy to them. Using these indicators, a score is calculated from 0 to 100, with 0 being the worst scenario and 100 the best. With the data obtained from the scores, Figure 1 was created, which makes it possible to identify how social and family networks have evolved in each South American country in the period 2007-2023, offering a panoramic view that makes it feasible to compare countries in this region of the world which, despite being a continent that shares several social and cultural characteristics, shows quite considerable differences with respect to the element analyzed.

Figure 1 shows marked differences between South American countries in this component. While Argentina consistently leads with the highest values, Peru is positioned as the country with the lowest scores, reflecting a lower perception of social and family support among its population. These discrepancies suggest the influence of cultural, economic and political factors that shape the social dynamics in each country.

To deepen the analysis, a table of statistics was used to identify significant variations between specific years and common patterns within the region. The stability observed in countries such as Argentina contrasts with fluctuations in other nations, highlighting the need for policies aimed at strengthening the social fabric in the most vulnerable communities.

### Interpersonal trust by country, period 2007 – 2023

The third key element of social capital, Interpersonal Trust, measures two indicators: the perception of trust in the immediate social environment and people’s willingness to selflessly help others. The data reveal that this dimension presents the lowest values among the analyzed elements of social capital, with scores that do not exceed 45 points on average at the regional level.

The series shows significant volatility in countries such as Ecuador and Paraguay, suggesting an unstable perception of social trust in these contexts. In Ecuador, the peak reached in 2009 contrasts with abrupt drops in subsequent years, linked to adverse economic and political events. Specifically, the economic recession that began in 2012, the fall in crude oil prices and the increase in public debt generated a climate of social discontent that derived in multiple protests and distrust towards institutions. ^58^

**Figure 2.**
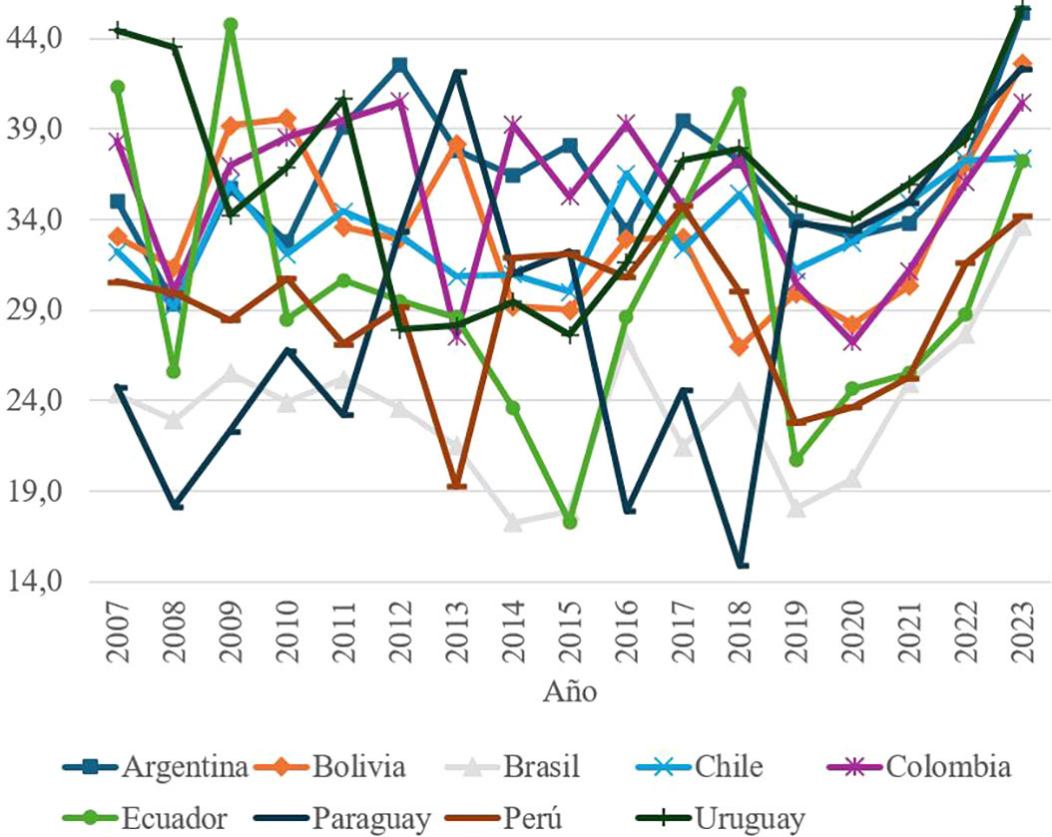
Interpersonal trust by country, 2007–2023.

These results, shown in Figure 2, highlight the need to promote policies that strengthen social cohesion and inter-individual trust, particularly in contexts of economic instability.

### Social and civic engagement by country, period 2007 – 2023

**Figure 3.**
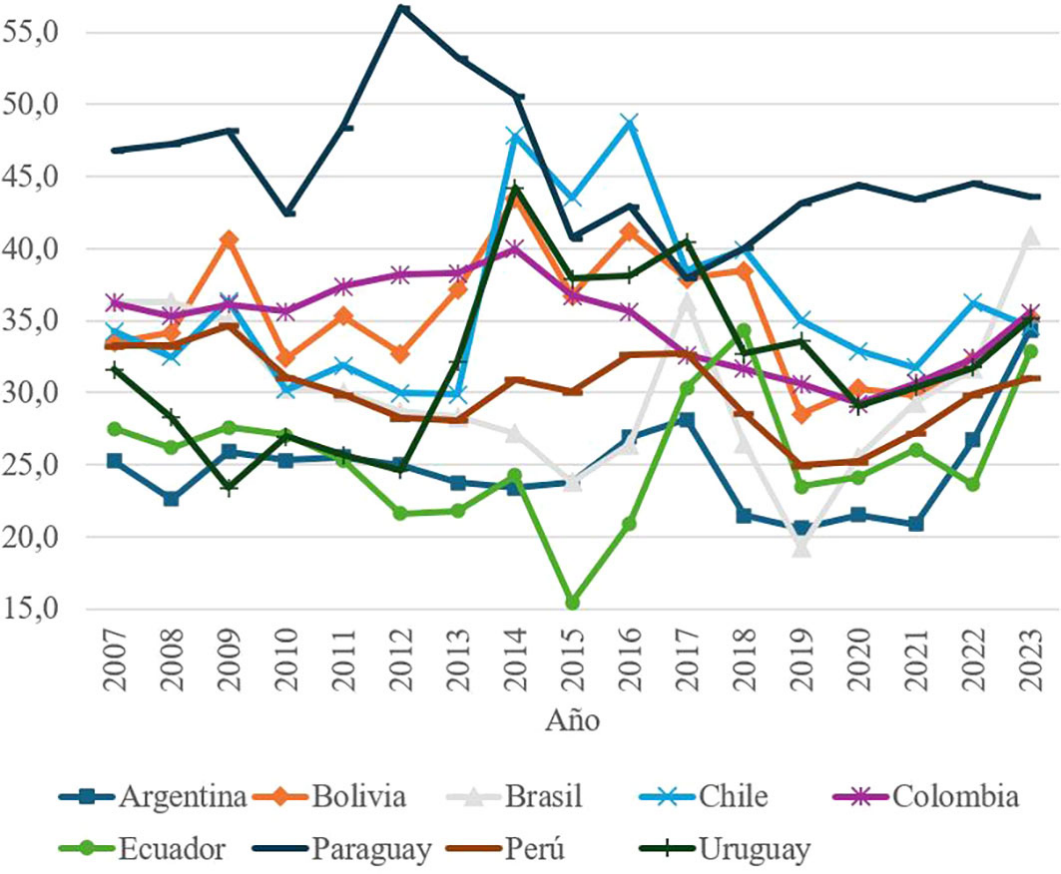
Civic and social participation by country, 2007–2023.

Social and civic participation, another fundamental element of social capital, includes indicators related to donating to charities, electoral registration, volunteering and expressing opinions to public officials. At the regional level, as shown in Figure 3 the scores are notably low, with an average of less than 55 points. In 2015, Ecuador presented the lowest value recorded (15.4 points), evidencing a critical lack of participation in civic activities. This problem is mainly based on the great inequalities that exist between inhabitants of the same country who, despite sharing several social and cultural characteristics, do not have the same opportunities. In this sense, each citizen is inserted into political life in a different way, in many cases guided by their life stories, their economic conditions, the level of education to which they had access and the influence of the social environment.

Low levels of participation are associated with structural inequalities, economic constraints and a low perception of political efficacy among citizens. In addition, factors such as the lack of volunteer training programs and recurrent economic crises aggravate this problem. The absence of government support for community initiatives limits the development of a culture of civic engagement in the region. ^59^

### Social tolerance by country, period 2007 – 2023

Social Tolerance, measured through indicators such as acceptance of ethnic groups, LGBT communities and migrants, shows a positive trend compared to other components of social capital. Uruguay consistently leads in this aspect, with scores that reflect high levels of inclusion and social cohesion.

Uruguay’s leadership in this dimension is explained by its strong middle class, low incidence of extreme poverty, and a progressive legislative framework that has strengthened the rights of historically discriminated groups. ^2^ These policies have allowed Uruguay to stand out as a model of equity and inclusion in South America.

**Figure 4.**
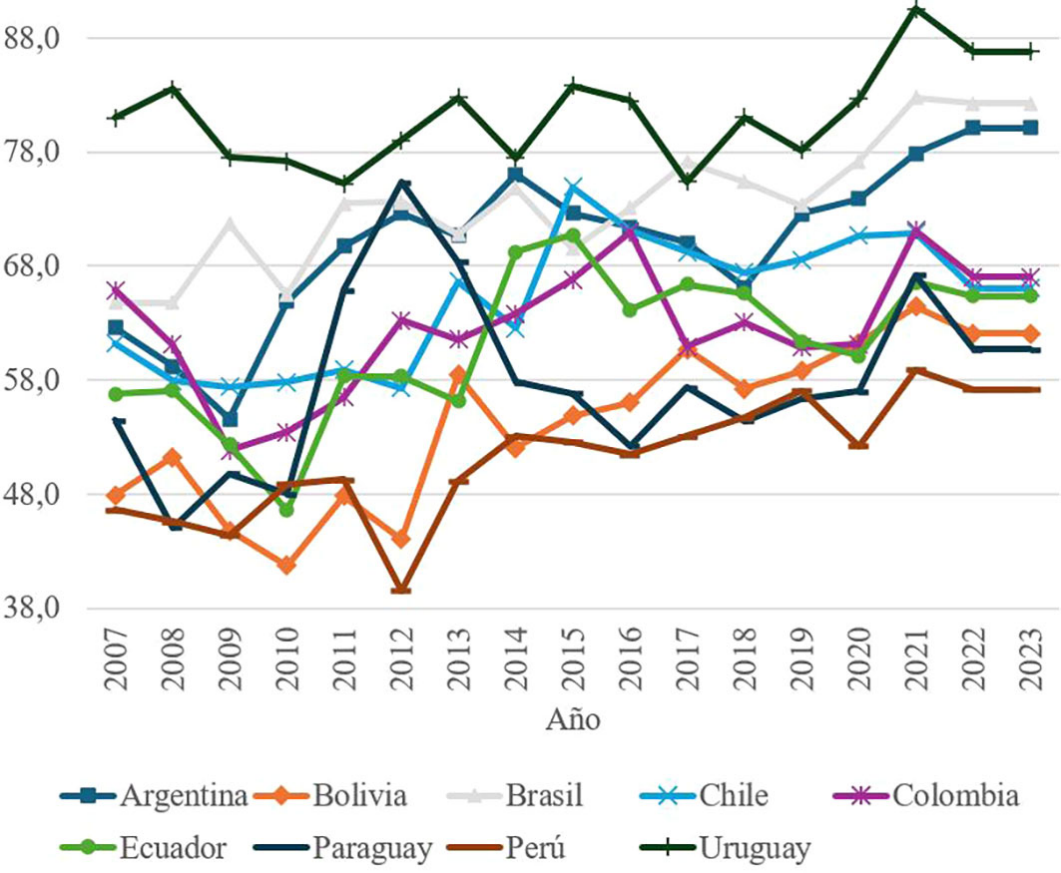
Social tolerance by country, 2007–2023.

An important achievement in this part of the world is characterized by nations with large socioeconomic gaps between social groups. In this regard, in recent years Uruguay has passed new legislation to provide protection and prominence to groups that have historically been discriminated against. The country is currently leading the region in the implementation of reforms that promote the rights of LBGT groups, citizens with disabilities and Afro-descendant ethnic groups, as shown in Figure 4.

### Results of the econometric analysis

The results in Table 1 the model presents a high explanatory power, with an R-squared of 0.9991, suggesting that almost all of the variability in the dependent variable is explained by the independent variables. Furthermore, the F(6,146) = 28019.62 test with a p-value = 0.0000 indicates that the model as a whole is highly significant. However, the high precision of the fit suggests the possibility of collinearity, which could be influencing the estimated coefficients.

**Table 1.** Durbin-Wu-Hausman test - Social network auxiliary regression.

Source|	SS	df	MS	Number of obs	153
				F(6, 146) = 28019.62	
Model|	3968.7923	6	661.465383	Prob > F = 0.0000	
Residual|	3.44665454	146	.023607223	R-squared = 0.9991	
				Adj R-squared = 0.9991	
Total|	3972.23895	152	26.133151	Root MSE = .15365	

social_networks	Coef.	Std. Err.	t	P>|t|	[95% Conf. Interval]
social_capital	4.994356	0150903	330.96	0.000	4.964532 5.024179
social_tolerance	-.9997837	.0037183	-268.89	0.000	-1.007132 -.9924352
social_civic_participation	-.9960573	.0037184	-267.88	0.000	-1.003406 -.9887086
interpersonal_trust	-1.000091	.0034442	-290.37	0.000	-1.006897 -.9932837
personal_family_relationships	-1.001549	.0044781	-223.66	0.000	-1.010399 -.9926986
economic_growth	-.0020643	.0022835	-0.90	0.367	-.0065773 .0024488
economic_growth	.4178443	.256199	1.63	0.105	-.0884934 .9241821

The analysis of Table 2 presented reveals a significant endogeneity problem in the estimation of the regression model. A first clear indication is the coefficient of determination, which implies that the model explains 100% of the variability in the dependent variable. The coefficient associated with the auxiliary regression residuals variable (Resid) is significant, indicating that the auxiliary regression residuals are highly correlated with the dependent variable, confirming the existence of endogeneity. In addition, the coefficients of the explanatory variables present extremely large values or close to their own confidence limits. The magnitude of the coefficients, especially that of “social capital” (18.16585) and the constant (_cons = 127.25), reinforces the suspicion that the model is misspecified.

**Table 2.** Main regression with the residual variable of the auxiliary regression.

Source|	SS	df	MS	Number of obs	153
				F(7, 145) = 99999.00	
Model|	5321.35765	7	760.19395	Prob > F = 0.0000	
Residual|	5.3380e-11	145	3.6814e-13	R-squared = 1.0000	
				Adj R-squared = 1.0000	
Total|	5321.35765	152	35.0089319	Root MSE = 6.1e-07	

economic_growth	Coef.	Std. Err.	t	P>|t|	[95% Conf. Interval]
personal_family_relationships	8.98e-08	3.28e-07	0.27	0.27	-5.58e-07 7.38e-07
social_networks	-8.637941	3.30e-07	-2.6e + 07	0.000	-8.637941 -8.63794
interpersonal_confidence	-3.881455	3.27e-07	-1.2e + 07	0.000	-3.881456 -3.881454
social_civic_participation	-3.280932	3.25e-07	-1.0e + 07	0.000	-3.280933 -3.280932
social_tolerance	-3.803154	3.27e-07	-1.2e + 07	0.000	-3.803155 -3.803153
social_capital	18.16585	1.63e-06	1.1e + 07	0.000	18.16585 18.16586
Residence	8.637941	7.81e-08	1.1e + 08	0.000	8.637941 8.637941
cons	127.25	1.00e-06	1.3e + 08	0.000	127.25 127.25

The problem of endogeneity in studies on social capital and economic growth is particularly relevant because many of the explanatory variables, such as social networks, interpersonal trust and social participation, can be simultaneously determined by economic factors. This generates a bidirectional causal relationship, where social capital influences economic growth, but also economic growth affects the formation of social capital.

In Table 3 the Ordinary Least Squares (OLS) model in its first stage presents a highly explanatory regression with an R-squared of 0.9991, indicating that the model explains 99.91% of the variability in the dependent variable. Furthermore, the adjusted R-squared value is the same, suggesting that the inclusion of the explanatory variables does not introduce overfitting in the regression. The F-statistic (28019.62) is extremely high, with an associated probability of 0.0000, indicating that, taken together, the explanatory variables have a significant impact on the dependent variable.

**Table 3.** Two-stage OLS development. Stage 1.

Source|	SS	df	MS	Number of obs	153
				F(6, 146) = 28019.62	
Model|	3968.7923	6	661.465383	Prob > F = 0.0000	
Residual|	3.44665454	146	.023607223	R-squared = 0.9991	
				Adj R-squared = 0.9991	
Total|	3972.23895	152	26.133151	Root MSE = .15365	

social_networks	Coef.	Std. Err.	t	P>|t|	[95% Conf. Interval]
social_capital	4.994356	.0150903	330.96	0.000	4.964532 5.024179
interpersonal_trust	-1.000091	.0034442	-290.37	0.000	-1.006897 -.9932837
personal_family_relationships	-1.001549	.0044781	-223.66	0.000	-1.010399 -.9926986
social_civic_participation	-.9960573	.0037184	-267.88	0.000	1.003406 -.9887086
social_tolerance	-3.803154	3.27e-07	-268.89	0.000	-1.007132 -.9924352
economic_growth	-.0020643	.0022835	-0.90	0.367	-.0065773 .0024488
economic_growth	.4178443	.256199	1.63	0.105	-.0884934 .9241821

Regarding the coefficients of the variables, it is observed that social capital has a positive coefficient of 4.994, suggesting that an increase in this variable is associated with a significant increase in the dependent variable. This result is statistically significant (p = 0.000), with a narrow confidence interval between 4.964 and 5.024, which reinforces the robustness of the finding.

On the other hand, variables such as interpersonal trust, family and personal relationships, civic social participation and social tolerance present negative and highly significant coefficients (p = 0.000). In particular, social tolerance has a coefficient of -3.803, which implies a strong negative effect on the dependent variable. This result could be interpreted to mean that certain forms of social interaction, when accompanied by distrust or closed relationships, can hinder economic growth or social development. Economic quality is not statistically significant (p = 0.367), suggesting that its effect on the dependent variable is inconclusive in this model.

Table 4 shows the OLS model which reports a statistic F(5, 147) = 5.21 with an associated probability of 0.0002, indicating that the set of explanatory variables has a significant joint effect on the dependent variable. However, the R-squared is 0.1506, suggesting that the model explains only 15.06% of the variability of the dependent variable. Although the adjusted R-squared (0.1217) is lower, it suggests that some variables contribute to the model, but the model still has low predictive power.

**Table 4.** Two-stage OLS development. Stage.

Source|	SS	df	MS	Number of obs	153
				F(5, 147) = 5.21	
Model|	801.196087	5	160.239217	Prob > F = 0.0002	
Residual|	4520.16156	147	30.7493984	R-squared = 0.1506	
				Adj R-squared = 0.1217	
Total|	5321.35765	152	35.0089319	Root MSE = 5.5452	

economic_growth	Coef.	Std. Err.	t	P>|t|	[95% Conf. Interval]
personal_family_relationships	.0602978	.088208	0.68	0.495	-.1140218 .2346174
interpersonal_confidence	-.1397144	.0703536	-1.99	0.049	-.2787495 -.0006793
social_civic_participation	.2184732	0620681	3.52	0.001	.0958121 .3411342
social_tolerance	.1124185	.0535748	2.10	0.038	.0065422 .2182949
social_networks_hat	-.3796057	.1044556	-3.63	0.000	-.5860344 -.173177
cons	64.8433	7.577112	8.56	0.000	49.86916 79.81744

Family and Personal Relationships has a positive coefficient of 0.0603, but its p-value (0.495) indicates that it is not statistically significant. This suggests that there is not enough evidence to affirm that this aspect of social capital influences the dependent variable.

For Interpersonal trust its coefficient is -0.1397, with a p-value of 0.049, indicating statistical significance at 5%. Since the confidence interval is completely in the negative range (-0.2787 to -0.0007), it can be concluded that higher interpersonal trust could be associated with lower economic growth in this context, which is contrary to standard theory and could be due to indirect effects or measurement problems.

The Civic Social Participation coefficient is 0.2185, with a p-value of 0.001, indicating a positive and statistically significant relationship. This suggests that a higher level of participation in civic social activities is associated with higher economic growth, aligning with the literature that highlights the importance of social cohesion and collaboration in economic development.

Social Tolerance has a positive coefficient of 0.1124 and a p-value of 0.038, suggesting a positive and significant relationship with economic growth. This finding reinforces the idea that more tolerant societies can generate higher levels of cooperation and development.

Social Networks (instrumentalized variable) presents a negative coefficient of -0.3796 with a p-value of 0.000, indicating a negative and significant relationship. This result is relevant, as it suggests that social networks (as measured in this model) may be associated with adverse effects on economic growth. Possible explanations include the use of social networks in non-productive activities or the effect of overconnectivity on the dilution of effective social capital.

This model suggests that certain aspects of social capital, such as civic participation and social tolerance, have a positive and significant effect on the dependent variable, while others, such as interpersonal trust and the use of social networks, seem to have negative effects in this particular context.

One of the main strengths of using instrumental variables and two-stage least squares models lies in their ability to address endogeneity problems, which are common in studies on social capital and economic growth, thereby contributing to more consistent estimators. Nevertheless, as with any econometric technique, this approach also has limitations. In particular, the results may be sensitive to the choice of instruments, which requires careful validation to ensure they meet the criteria of relevance and exogeneity. Despite this, the methodology applied is appropriate for the objectives of this study and provides robust evidence on the relationship under analysis.

## Discussions

CS has been widely discussed as a determining factor in the processes of economic growth and development. However, its impact is not homogeneous and varies according to the institutional, social and productive structures in which it is inserted. From a structuralist perspective, economic development does not arise exclusively from the accumulation of productive factors or individual behavior, but from the transformation of pre-existing structures that determine access to opportunities and the distribution of economic power. ^60, 61^

From an institutionalist viewpoint, ^60^ emphasizes that formal and informal institutions play a key role in reducing uncertainty and generating incentives for investment and growth. In this sense, civic participation contributes to the creation of structures that facilitate the implementation of long-term policies and economic stability. ^62^ Warn that, in some contexts, participation within closed networks can reinforce structures of exclusion and clientelism, limiting social mobility and distorting resource allocation mechanisms.

One of the most discussed aspects of social capital theory is the role of interpersonal trust in economic development ^28^ argues that societies with high levels of trust show greater efficiency in cooperation and lower transaction costs, which facilitates economic activity and investment in long-term projects. From a structuralist point of view, however, this argument has been questioned ^63^ warns of the existence of “perverse” social capital, i.e., networks of trust that can foster exclusion, cronyism and clientelism. In economies with a poorly diversified productive structure, interpersonal trust can consolidate economic relationships based on personal loyalty, instead of promoting competition and efficiency. This phenomenon can also be analyzed in the light of ^64^ theory of delayed industrialization, which argues that in certain contexts trust within closed groups can act as a barrier to productive modernization. In contrast, some authors argue that interpersonal trust is not the problem in itself, but its interaction with weak institutional structures.

The role of social networks in economics has been widely discussed in the structuralist and neo institutionalist literature. Since ^65^ theory, it has been argued that well-structured social networks can facilitate access to valuable information, promote social mobility and improve resource allocation. However, when these networks are too closed, they can generate exclusionary structures that limit competition and innovation. From an institutionalist perspective, ^66^ argue that in economies where extractive institutions predominate, social networks can become a control mechanism that perpetuates inequalities and restricts the participation of external actors in economic activity. In this sense, the impact of social networks is neither intrinsically positive nor negative, but depends on the institutional conditions in which they operate.

From ^17^ perspective, the family can be an important source of social capital, but its impact on economic development depends on its capacity to generate access to wider networks. In societies where family relationships play a central role in the social structure, family social capital can become a relevant economic resource. However, ^61^ dependency theory argues that in peripheral economies, family relations alone are not sufficient to modify economic structures marked by inequality and external dependence. In these contexts, the social capital generated in the family environment can be a limited resource if it is not articulated with institutions that allow its conversion into concrete economic opportunities.

## Conclusions

Social capital (SC) has proven to be a determining factor in economic growth and development processes, although its impact is not uniform and depends on the institutional, social and productive structures in which it is embedded. Evidence suggests that, in contexts with strong and inclusive institutions, CS can reduce uncertainty, facilitate cooperation and improve resource allocation by reducing transaction costs. However, its impact can become negative when it operates in economies with poorly diversified productive structures and extractive institutions, as it tends to consolidate relationships based on personal loyalty and clientelism, restricting competition and social mobility. In this sense, rather than an intrinsically positive or negative asset, the SC acts as an amplifier of pre-existing institutional conditions.

From an institutionalist perspective, the interaction between social networks and the regulatory framework is key to determining whether the SC facilitates innovation and growth or, on the contrary, reinforces structures of exclusion. In economies with excessively closed networks, interpersonal trust can become an exclusionary mechanism, hindering the incorporation of new actors and limiting productive modernization. Thus, the impact of CS depends not only on its presence, but also on its integration with institutions that guarantee equitable access to economic opportunities and promote fair competition. This implies that its effect on economic development cannot be analyzed in isolation, but rather within an institutional ecosystem that determines its incentives and restrictions.

To enhance the positive effects of SC on economic development, public policies should focus on the creation of inclusive institutions that promote equitable participation in the economy and reduce the risks of capture by closed networks. This includes strengthening transparency mechanisms, regulating conflicts of interest and promoting legal frameworks that encourage competition and innovation. It is also crucial to promote education and training policies that expand opportunities for access to high-value networks, preventing SC from being limited to exclusive circles. As future lines of research, we recommend exploring the role of CS in the digitalization of the economy, its relationship with the automation of work and its impact on the sustainability of development, as well as methodologies to measure its effect in different institutional and productive contexts.

## Ethical approval

Ethical approval was not required for this study as it used publicly available, anonymized secondary data from the Legatum Prosperity Index. The study was conducted in accordance with the Declaration of Helsinki.

## Data Availability

The data underlying the results presented in this article were obtained from an external source: the Legatum Institute’s Prosperity Index. The dataset is publicly available and can be accessed at the following link: https://index.prosperity.com/about-prosperity/prosperity-index . ^57^ No proprietary or original data were generated or collected by the authors for this study.
