# Supplementary material for: Social Capital and Economic Growth: An Analysis in South America
Source: F1000Res. 2025 Sep 8;14:797. Originally published 2025 Aug 18. [Version 2] doi: 10.12688/f1000research.166766.2 (PMC13148854; doi:10.12688/f1000research.166766.2)
Supplement: Peer review history

# Reviewer report: Reviewer response for version 2

**DOI:** 10.5256/f1000research.187604.r407045

**Published:** 2026-01-28

**Authors:** Mauricio Carvache-Franco

**Affiliations:** 1 Facultad de Turismo y Hotelería, Bolivarian University of Ecuador, Duran, Guayas, 092301, Ecuador

The article has a good structure and is robust in all sections. Its well-structured literature review strengthens the article. The methodology is appropriate. The results are as indicated and accurately present the study's findings. The discussion is well-written. It is recommended to expand on the methodology, particularly regarding how the sample was selected.

Is the work clearly and accurately presented and does it cite the current literature?

Yes

If applicable, is the statistical analysis and its interpretation appropriate?

Yes

Are all the source data underlying the results available to ensure full reproducibility?

Yes

Is the study design appropriate and is the work technically sound?

Yes

Are the conclusions drawn adequately supported by the results?

Yes

Are sufficient details of methods and analysis provided to allow replication by others?

Yes

Reviewer Expertise:

Administración

I confirm that I have read this submission and believe that I have an appropriate level of expertise to confirm that it is of an acceptable scientific standard.

# Reviewer report: Reviewer response for version 2

**DOI:** 10.5256/f1000research.187604.r412226

**Published:** 2025-11-13

**Authors:** Ruth Zamora-Sánchez

**Affiliations:** 1 University of the Basque Country, Bilbao, Basque Country, Spain

The paper presents an original approach within its field of study by emphasizing stakeholders rather than focusing solely on direct outcome measurements. Moreover, it reflects a solid command of the existing body of literature, drawing on a wide range of sources that encompass foundational studies, recent contributions, and pertinent theoretical perspectives. There is no indication that any major works have been omitted.

 The methodology section is described in considerable detail.

 The results are communicated clearly and analyzed appropriately, and the conclusions effectively integrate and synthesize the key components of the paper.

Is the work clearly and accurately presented and does it cite the current literature?

Yes

If applicable, is the statistical analysis and its interpretation appropriate?

Yes

Are all the source data underlying the results available to ensure full reproducibility?

Yes

Is the study design appropriate and is the work technically sound?

Yes

Are the conclusions drawn adequately supported by the results?

Yes

Are sufficient details of methods and analysis provided to allow replication by others?

Partly

Reviewer Expertise:

Social capital, Business Administration, Economic development, Entrepreneurship, Education, Gamification.

I confirm that I have read this submission and believe that I have an appropriate level of expertise to confirm that it is of an acceptable scientific standard.

# Reviewer report: Reviewer response for version 1

**DOI:** 10.5256/f1000research.183805.r407039

**Published:** 2025-08-29

**Authors:** Ruth Zamora-Sánchez

**Affiliations:** 1 University of the Basque Country, Bilbao, Basque Country, Spain

The approach of the paper is original in the area of study, since it focuses on the stakeholders instead of the direct measurement of results. In adittion, the paper effectively demonstrates a strong understanding of the relevant literature in the field. The author cites a comprehensive range of sources, including seminal works, recent research, and relevant theoretical frameworks. There is no evidence of any significant work being overlooked.

 The methodology is explained quite extensively. However, the importance of the methodology used and why it is used, advantages and disadvantages should be discussed. How the sample was selected.

 The results are presented clearly and analyzed appropriately. The conclusions adequately tie together the other elements of the paper.

Is the work clearly and accurately presented and does it cite the current literature?

Yes

If applicable, is the statistical analysis and its interpretation appropriate?

Yes

Are all the source data underlying the results available to ensure full reproducibility?

Yes

Is the study design appropriate and is the work technically sound?

Yes

Are the conclusions drawn adequately supported by the results?

Yes

Are sufficient details of methods and analysis provided to allow replication by others?

Partly

Reviewer Expertise:

Social capital, Business Administration, Economic development, Entrepreneurship, Education, Gamification.

I confirm that I have read this submission and believe that I have an appropriate level of expertise to confirm that it is of an acceptable scientific standard.
